# Exposure to Aerosolized Algal Toxins in South Florida Increases Short- and Long-Term Health Risk in *Drosophila* Model of Aging

**DOI:** 10.3390/toxins12120787

**Published:** 2020-12-11

**Authors:** Jiaming Hu, Jiaqi Liu, Yi Zhu, Zoraida Diaz-Perez, Michael Sheridan, Haley Royer, Raymond Leibensperger, Daniela Maizel, Larry Brand, Kimberly J. Popendorf, Cassandra J. Gaston, R. Grace Zhai

**Affiliations:** 1 Rosenstiel School of Marine and Atmospheric Science, University of Miami, Miami, FL 33149, USA; jxh1011@miami.edu (J.H.); mts125@miami.edu (M.S.); haley.royer@rsmas.miami.edu (H.R.); rxl652@miami.edu (R.L.III); cgaston@rsmas.miami.edu (C.J.G.); 2 Programs in Biomedical Sciences, University of Miami Miller School of Medicine, Miami, FL 33136, USA; 3 Department of Molecular and Cellular Pharmacology, University of Miami Miller School of Medicine, Miami, FL 33136, USA; jxl2626@miami.edu (J.L.); y.zhu17@med.miami.edu (Y.Z.); zdiaz@med.miami.edu (Z.D.-P.); 4 Department of Ocean Sciences, Rosenstiel School of Marine and Atmospheric Science, University of Miami, Miami, FL 33149, USA; dxm1457@rsmas.miami.edu (D.M.); kpopendorf@rsmas.miami.edu (K.J.P.); 5 Department of Marine Biology and Ecology, Rosenstiel School of Marine and Atmospheric Science, University of Miami, Miami, FL 33149, USA; lbrand@rsmas.miami.edu

**Keywords:** fresh water algal blooms, blue-green algae, cyanobacteria, aerosol toxins, South Florida

## Abstract

Harmful algal blooms (HABs) are a rising health and environmental concern in the United States, particularly in South Florida. Skin contact and the ingestion of contaminated water or fish and other seafood have been proven to have severe toxicity to humans in some cases. However, the impact of aerosolized HAB toxins is poorly understood. In particular, knowledge regarding either the immediate or long-term effects of exposure to aerosolized cyanotoxins produced by freshwater blue-green algae does not exist. The aim of this study was to probe the toxicity of aerosolized cyanobacterial blooms using *Drosophila melanogaster* as an animal model. The exposure of aerosolized HABs at an early age leads to the most severe long-term impact on health and longevity among all age groups. Young groups and old males showed a strong acute response to HAB exposure. In addition, brain morphological analysis using fluorescence imaging reveals significant indications of brain degeneration in females exposed to aerosolized HABs in early or late stages. These results indicate that one-time exposure to aerosolized HAB particles causes a significant health risk, both immediately and in the long-term. Interestingly, age at the time of exposure plays an important role in the specific nature of the impact of aerosol HABs. As BMAA and microcystin have been found to be the significant toxins in cyanobacteria, the concentration of both toxins in the water and aerosols was examined. BMAA and microcystin are consistently detected in HAB waters, although their concentrations do not always correlate with the severity of the health impact, suggesting the potential contribution from additional toxins present in the aerosolized HAB. This study demonstrates, for the first time, the health risk of exposure to aerosolized HAB, and further highlights the critical need and importance of understanding the toxicity of aerosolized cyanobacteria HAB particles and determining the immediate and long-term health impacts of HAB exposure.

## 1. Introduction

Harmful algal blooms (HABs) produce a variety of toxins [1,2]. These toxins can accumulate in shellfish, fish, and other marine organisms, thus causing harm, directly or indirectly, to animals, humans, and marine and freshwater ecosystems [1,3]. They can potentially cause major economic loss to aquaculture, fisheries, and tourism operations as well [3]. During the past three decades, the frequency, intensity, and global distribution of HABs have largely increased in the ocean, nearshore coastal waters, and freshwater ecosystems [1,4]. HABs have been found in all 50 states in the USA, from large freshwater lakes, such as the Great Lakes to smaller inland lakes, rivers, marine coastal areas, and estuaries [5]. Just as in many other parts of the USA, many southern Florida waters have blooms of cyanobacteria as well, including Lake Okeechobee and the St. Lucie and Caloosahatchee rivers and estuaries [6]. 

Cyanobacteria produce a wide array of toxins, such as microcystin, β-N-methylamino-L-alanine (BMAA), nodularin, cylindrospermopsin, saxitoxin, and anatoxins [6,7]. These naturally produced toxins can potentially have toxic or harmful effects on human beings and other organisms through various routes of exposure, such as skin contact, inhalation, hemodialysis, and ingestion of contaminated drinking water or fish [7,8]. In these exposure pathways, skin contact and ingestion have been the main mechanisms for direct toxicity to humans, and thus cause individual poisoning due to recreational activities in rivers or lakes with cyanobacteria blooms [9]. While it has been shown that people can be exposed to HABs by breathing in tiny airborne droplets from a contaminated water body, and inhalation is one of the possible exposure routes to cause human illnesses and diseases [10], little is known about the exact amount of toxin transfer between air and water, and their direct short- and long-term effects on human health through inhalation exposure. Among the naturally produced cyanobacterial toxins, microcystins, a group of hepatotoxins that specifically target the liver and can lead to death in wildlife [10,11,12,13], and BMAA, a neurotoxin that leads to several severe neurodegenerative diseases [6,14,15], are of particular concern in this study due to their serious health impacts reported in previous studies.

The fruit fly *Drosophila melanogaster* is a widely used animal model that offers several important advantages for human health studies, including a short life cycle and rich genetic resources [16]. *Drosophila* has been shown to be an ideal animal model to measure coordinated motor functions as they exhibit several highly stereotypical and measurable behaviors that reflect animal fitness and the integrity of nervous system [17,18,19]. Among them, the negative geotaxis response, first described by Seymour Benzer [20], is the most widely used. The negative geotaxis response of flies shows an age-dependent decline [21], and is found to be impaired in many *Drosophila* models of human neurological disorders, such as tauopathy [22], Huntington’s disease [23], Parkinson’s disease [24], and spinocerebellar ataxia [25]. *Drosophila* is also an optimal model for aging studies because it has a relatively short lifespan, with a maximum between 90 to 100 days [26,27,28]. Due to its relatively short biological life cycle, it is possible to address the longitudinal effects of early exposure to aerosolized cyanobacterial toxicity, through development and adulthood. The comprehensive toxicity of dietary intake of BMAA has been found to reduce fruit flies’ locomotor function and shorten their life spans [26]; however, the impact of aerosolized cyanobacterial toxins had not yet been studied.

The diversity of these traits makes the fruit fly an excellent research organism for us to learn the molecular mechanisms underlying development, aging, behavior, and neurodegenerative disease [16,29]. Here, *Drosophila* has been used as an animal model system to access the acute and long-term impacts following exposure to aerosols generated from different water sources, taking advantage of the relatively high throughput of examining a large number of fruit flies at the same time, which is particularly suitable for assessing the impact of environment interaction and on aspects of behavior, aging, and human health.

## 2. Results

### 2.1. Aerosolized HABs Impairs Drosophila Locomotor Functions

**Figure 1:**
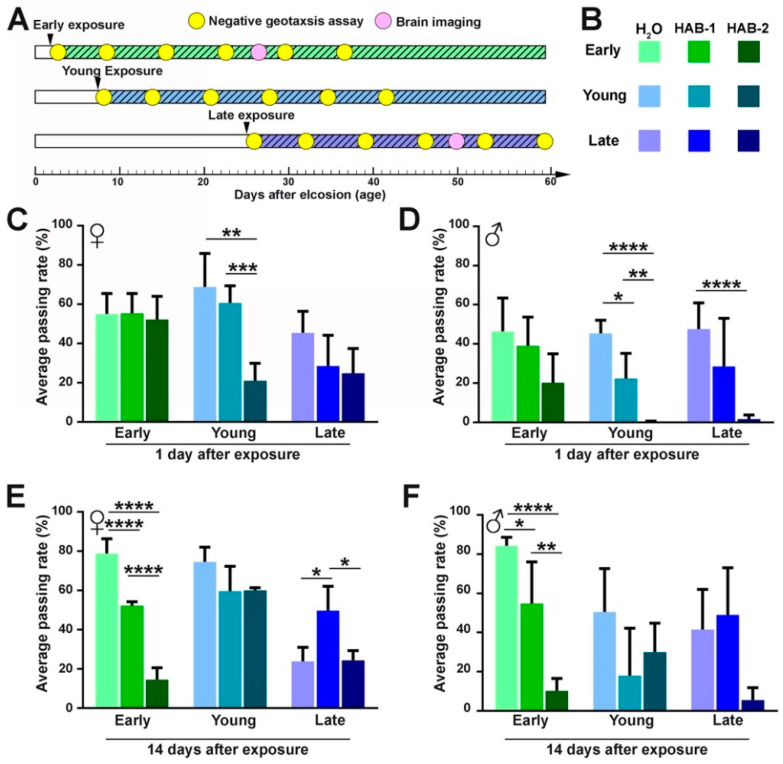
Aerosolized HABs impairs *Drosophila* locomotor functions. (**A**) Experimental protocol. Dates of exposure were marked by triangles. Dates of behavior and brain imaging analyses are indicated by circles. (**B**) Legend of negative geotaxis assay results. (**C**,**D**) The female and male negative geotaxis assay performed one day after exposure. (**E**,**F**) The female and male negative geotaxis assay performed 14 days after exposure. Approximately 100 flies were tested for each group. All data are presented as mean ± S.D. *n* = 10. The significance level of C-F was analyzed by one-way analysis of variance (ANOVA) followed by post hoc Tukey test. * *p* ≤ 0.05, ** *p* ≤ 0.01, *** *p* ≤ 0.001, **** *p* ≤ 0.0001.

To examine the immediate and the long-term effect of aerosolized HABs, the negative geotaxis assays were performed at 1, 7, 14, 21, 28, and 35 days after exposure (Figure 1A). One day after exposure was considered as immediate response and 14 days and beyond after exposure were considered as long-term response. For immediate response (Figure 1C,D), early exposure to HAB aerosols had no significant change in behavior one day after exposure. However, a significant reduction in behavior was detected in the young and the late exposure group immediately after exposure. In particular, young groups and late-stage males that were exposed to HAB-2 showed poorer climbing performance than those exposed to HAB-1 or water, suggesting that HAB-2 caused a more severe acute effect. When flies were examined at 14 days after exposure (Figure 1E,F), the early-stage groups exposed to HABs showed a significant decrease in climbing performance, compared to water and HAB-1. However, the young and the late-stage groups showed varying levels of recovery. These results suggest that the age of exposure is a critical determinant of the specificity of the impact. 

### 2.2. Exposure to Aerosolized HABs Causes Long-Term Detriment in Neuromuscular Functions

**Figure 2:**
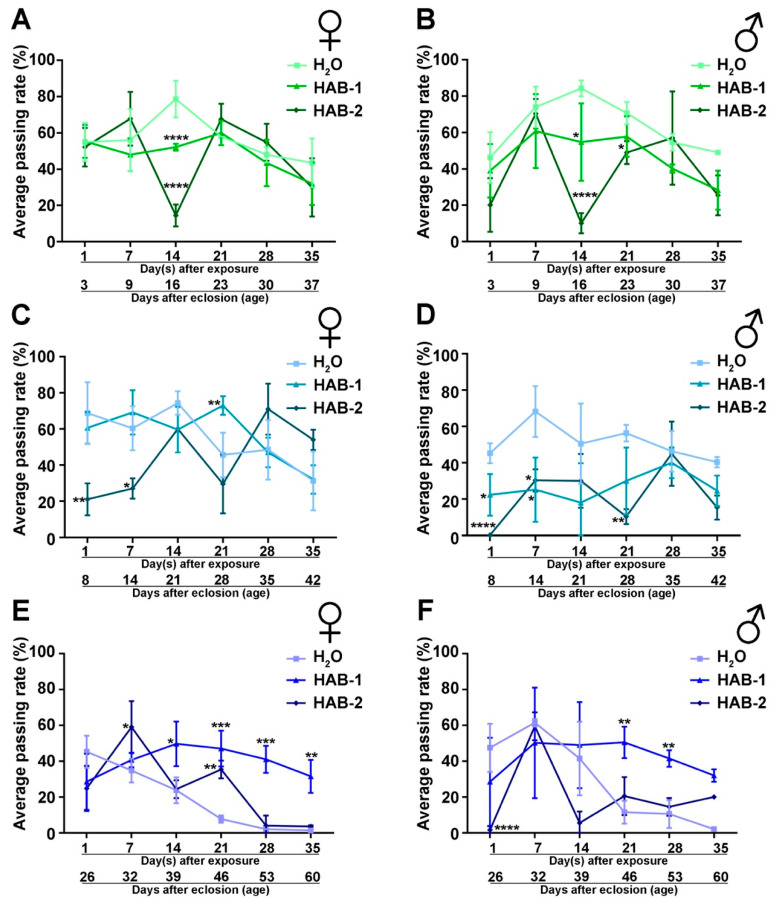
Exposure to aerosolized HABs causes long-term detriment in neuromuscular functions. Longitudinal climbing performance testing of female and male flies exposed to HAB at early-stage (**A**,**B**), at young-stage (**C**,**D**), and at late-stage (**E**,**F**). The significance level of (**C**–**F**) was analyzed by one-way analysis of variance (ANOVA) followed by post hoc Tukey test. * *p* ≤ 0.05, ** *p* ≤ 0.01, *** *p* ≤ 0.001, **** *p* ≤ 0.0001.

To uncover any potential recovery and further examine the long-term impact of exposure, a longitudinal behavior test was carried out to analyze the climbing performance at 1, 7, 14, 21, 28, and 35 days after exposure. Flies that were exposed to HAB aerosols at an early age showed a significant decline in climbing performance at 14 days after exposure (Figure 2A,B). Flies that were exposed at a young age showed a decline in climbing performance immediately, at 1 day and 7 days after exposure (Figure 2C,D). Notably, gender-specific differences were observed in the longitudinal analysis. For example, females exposed to HAB-1 at young age showed significantly higher climbing performance than those exposed to water at 21 days after exposure (Figure 2C). On the other hand, males exposed to both HABs at a young age had significantly lower climbing performance than the groups exposed to water (Figure 2D). A similar gender-specific difference was also observed in flies exposed to HABs at late stage, as females exposed to HABs at late stage showed significantly higher climbing performance than those exposed to water at multiple time points after exposure (Figure 2E). However, males exposed to HAB at late stage had a significant reduction in climbing ability immediately (1 day) after exposure. Late-stage males exposed to HAB-1 were doing significantly better than those exposed to water at 21 and 28 days after exposure (Figure 2F), suggesting that exposure to aerosolized HABs at late stage did not result in significant long-term damage compared to water exposure.

These results indicate that the exposure to aerosolized HABs at an early age showed the most detrimental long-term impact on fitness. Exposure at young- and late-stages showed the most immediate impact, but they were able to recover slightly and, therefore, showed less long-term impacts. Importantly, gender difference was observed where males were more vulnerable to immediate impact. These results revealed the age-dependent and gender vulnerability in the behavioral fitness after aerosolized HAB exposure. 

### 2.3. Early Exposure to HABs Particularly Reduces the Longevity of Drosophila 

**Figure 3:**
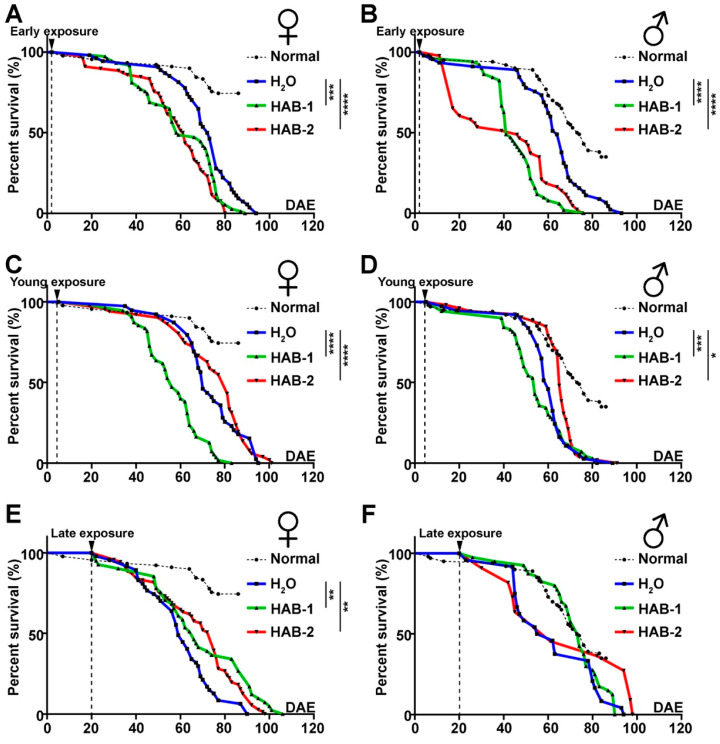
Exposure to aerosolized HABs reduces the longevity of *Drosophila*. Life span survival curve of female and male flies exposed to HAB at early-stage (**A**,**B**), at young-stage (**C**,**D**), and at late-stage (**E**,**F**). The normal fly lifespan (shown in black dashes) was used to make a comparison with the control group and experimental groups that were exposed to HAB water. The normal fruit fly was defined as flies cultured in the lab with sufficient food and water and without any other outside factors affecting them. They were provided with the same temperature, food, and were raised under the same light source. About 100 flies were tested in each age group with about 50 females and 50 males included. The significance level of all data was analyzed by the log-rank test. * *p* ≤ 0.05, ** *p* ≤ 0.01, *** *p* ≤ 0.001, **** *p* ≤ 0.0001.

In order to examine the impact of toxicity of aerosolized HAB particles on lifespan, the number of live and dead fruit flies were recorded daily since the first day of exposure. Flies exposed to either HAB at early-stage had a significantly reduced lifespan, with reduced survival rate evident as early as 10 days after exposure (Figure 3A,B). Flies exposed to HAB-1 at a young age showed significantly shortened life span (Figure 3C,D). HAB exposure at late stage did not significantly impact life span compared to water exposure (Figure 3E,F). These results indicate the age-dependent vulnerability to the exposure of aerosolized HABs, as early age exposure resulted in the most detrimental impact on the longevity and life span. 

### 2.4. Aerosolized HAB Significantly Impairs Drosophila Synapse Function

The nervous system of the fruit fly serves as a beneficial model for studying various aspects of human brain functions and degenerative diseases [30]. In order to address the association between neuropathological changes in the brain and the presence of aerosolized cyanotoxins, such as microcystin and BMAA, the brain morphology of early- and late-stage groups of *Drosophila* exposed to H_2_O and HAB-3 water were examined and analyzed. The brain dissection was done around 25 days after flies were exposed to each water source for 2 h (Figure 1A). The 25-day post-exposure time point was selected because this is the time point of 50% survival rate after exposure, suggesting a negative impact on viability and a likely presence of brain morphological phenotypes. 

**Figure 4:**
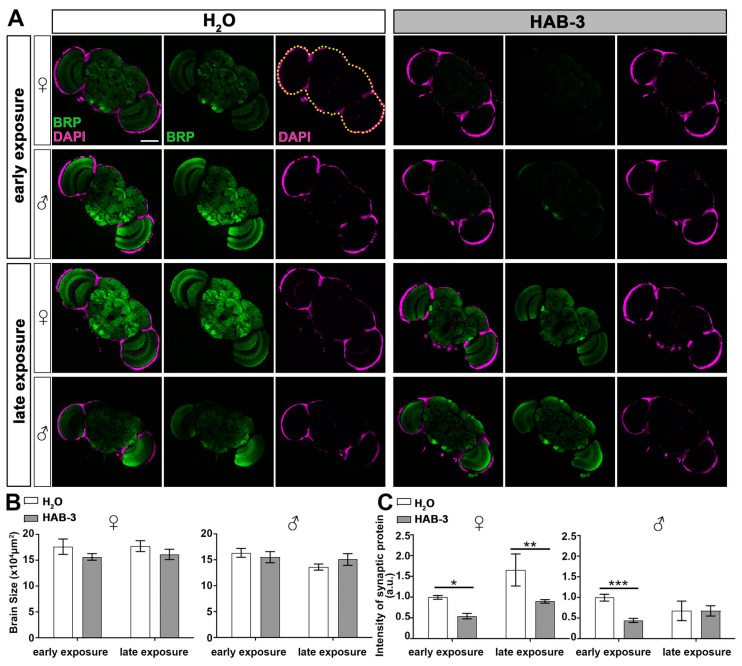
Aerosolized HABs significantly impair *Drosophila* synapse function. (**A**) Brains of early and late flies exposed to H_2_O and HAB-2 were probed for BRP (green) and DAPI (magenta). The yellow dashed line indicates the boundary of brain size. Representative scale bar: 100 μm. (**B**) Quantification of brain size. (**C**) Quantification of Brp intensity. All data are presented as mean ± S.D. The significance level was established by one-way ANOVA followed by post hoc Tukey test. * *p* ≤ 0.05, ** *p* ≤ 0.01, *** *p* ≤ 0.001.

Using quantitative fluorescence imaging analysis, brain size and synaptic structure were analyzed. Brain size was analyzed by using the volume of the nuclei marker DAPI. The synapse structure was analyzed by specific labeling with synaptic protein BRP. BRP is a cytomatrix protein that participates in the formation and maintenance of the active zone, promotes the clustering of calcium channels in the presynaptic membrane, and affects the efficacy of neurotransmission [31,32]. BRP levels directly correlated with the structural and functional integrity of synapses, and its reduction indicates neurodegeneration [33]. Although we did not find a significant decrease in brain size upon HAB exposure (Figure 4A,B), a remarkable reduction in the BRP fluorescence intensity was observed in the brains of the flies (both genders) exposed to HAB-3 at early-stage (Figure 4A,C). The brains of female flies exposed at late-stage also showed a significant decrease (Figure 4C). 

These results further indicate the vulnerability of early exposure. Consistent with the behavior and lifespan phenotypes, flies exposed to HABs at an early age have the most severe synaptic degeneration at 25 days after exposure. Furthermore, females are more vulnerable to brain degeneration compared to males when exposed to HABs at the late stage. These results reveal the long-term impact on the brain and highlight the risk of developing neurodegeneration upon exposure to HAB aerosols. 

### 2.5. BMAA and Microcystin Are Consistent Toxic Constituents of HABs

The chemical characteristics of the three HAB water sources are provided in Table 1. At the time of the bubbling experiments, the measured concentrations of cyanobacterial toxins in the water were 0.37 μg/L microcystin and 7.2 ng/L BMAA in HAB-1 (an environmental water sample), 0.01 μg/L microcystin and 1.7 ng/L BMAA in HAB-2 (environmental water sample), and 379.38 μg/L microcystin in HAB-3 (a laboratory-maintained monospecific culture of *Microcystis aeruginosa*; BMAA concentration was not analyzed). The aerosol microcystin concentration was below the limit of detection for HAB-1 and HAB-2, and the aerosol microcystin concentration was 62 pg/L for HAB-3. Microcystin (MC) is a class of heptapeptide compounds with congeners named to represent the standard abbreviations for the amino acids at positions two and four in the heptapeptide ring: leucine (L), alanine (A), phenylalanine (F), arginine (R), tryptophan (W), and tyrosine (Y); D-Asp indicates demethylation of the third moiety D-erythro-β-methylaspartic acid. The MC congeners present in the environmental water samples were predominantly MC-LR, with smaller amounts of MC-LA, MC-LY, and D-Asp-MC-LR (in HAB-1 congeners were 71%, 13%, 9%, and 3% MC-LR, MC-LY, D-Asp-MC-LR, and MC-LA, respectively, with MC-LW, MC-LF, and MC-YR present at less than 2% total MC; in HAB-2, congeners were 70%, 19%, and 11% MC-LR, MC-LA, and MC-LY, respectively). The MC congeners present in the monospecific culture sample (HAB-3) were predominantly MC-LR as well (78%), with MC-LF, MC-LW, and MC-LY (6%, 6%, and 6%, respectively), with 3% or less D-Asp-MC-LR and MC-YR. In the HAB aerosols from the monospecific culture (HAB-3) the relative congener abundances differed from the water, with the aerosol MC congeners being 60% MC-LF, 29% MC-LR, and 11% MC-LW, showing similar differences between water and air congener distributions as previously observed [34,35].

## 3. Discussion

The impact of aerosolized cyanobacterial toxins on health using *Drosophila* as a model organism was examined in this study. The results showed that exposure to HABs impairs locomotor functions, shortens the life span, and causes *Drosophila* synapse dysfunction. Cyanotoxins, such as hepatotoxins, have the ability to cause long-term effects with chronic exposure [36]. We found in this study that aerosolized cyanobacterial HABs, containing toxins, such as BMAA and microcystin, have the ability to cause short-term and long-term impact on the health of different age groups. 

### 3.1. Age at Exposure Is a Major Determinant of the Health Impact of Aerosolized HABs 

Age played an important role in the effects of aerosolized HAB toxins. Exposure to aerosolized HABs at an early age reduced locomotor behavior resulting in a long-term impact, whereas exposure at later ages had the most immediate impact in neuromuscular functions. HAB aerosols reduced lifespan at all age stages. In particular, early age exposure showed the most significant impact of aerosolized HABs on longevity and health. Early exposure of aerosolized HAB toxins also showed remarkably impaired synapse integrity. These results indicate that exposure to aerosolized HABs at an early age has the most severe long-term impact on behavioral function, longevity, and neurodegeneration. Young and late stages were able to recover to certain extent from aerosolized HAB exposure and, therefore, showed less of a long-term impact, suggesting increased resilience in older age. A previous study in mice reported the age-dependent effects after oral route of exposure of microcystin-LR, and further suggested that the effects may be due to the differences in the amount of microcystin-LR uptake and also the age-dependent ability to detoxify the toxins in mice [12,37]. The different impact among age groups of *Drosophila* exposure to aerosolized HABs could also be due to the age-related ability of flies to detoxify or excrete aerosolized toxins since the early-stage groups were the most vulnerable to long-term HAB exposure in terms of locomotor functions, longevity, and synapse dysfunction in comparison with the other two tested age groups. Even though the locomotor functions of elder groups were reduced immediately after HAB exposure, they have more ability to recover from inhalation of aerosol HABs. Our studies using in vivo models are the first to identify age-dependent resiliency factors and pinpoint the high-risk groups that are most vulnerable to the exposure to aerosolized HABs.

### 3.2. Vulnerability of Gender Differences

Gender-specificity has been studied in many human neurological disorders, such as Alzheimer’s disease (AD) [38] and Parkinson’s disease [39]. It has been shown that women have significantly higher incidence rates of having any dementia and AD than men after 80 years old [38]. It has also been reported that adult female medaka fish have a higher susceptibility to chronic exposure to pure microcystin-LR or complex *Microcystis aeruginosa* extracts compared to males at both the cellular and the molecular levels [40]. In this study, females at late exposure were more vulnerable to brain degeneration than males, which suggests a gender vulnerability consistent with observations in other models [38,40]. The neurodegenerative phenotypes observed after long-term exposure to aerosolized HABs was significantly higher in older females than in older males, suggesting that senior females were more vulnerable to toxic responses and were more likely to have neurodegenerative issues than senior males. However, females were also observed to have significantly better climbing performance than males after HAB exposure, which indicates that the impact of exposure to aerosolized HABs is gender-specific and its vulnerability is associated with different behaviors and neurological disorders.

### 3.3. Contribution of BMAA and Microcystin to the Aerosol Toxicity

Both BMAA and microcystin were detected in the environmental water samples from which aerosols were generated for *Drosophila* exposures; in the aerosol phase microcystin was quantified from the high toxin concentration monospecific HAB culture. While BMAA and MC were not detected in the aerosols from environmental water samples, they may have been present at levels below the method limit of detection. In addition, for both the environmental water samples and monospecific cultures the aerosols generated represented particles produced from a complex water matrix that includes a wide range of algae-derived organic material as well as other diverse compound classes, thus the aerosols generated exposed the *Drosophila* to a potentially wide range of chemical constituents. The results in all cases were evaluated relative to a control experiment where *Drosophila* were exposed to aerosols generated from highly purified water (MilliQ 18 MΩ resistance water) to differentiate the impact of chemical exposure from the effects of the aerosolization experimental design.

Previously, the *Drosophila* model has been successfully established and the long-term and transgenerational effects of one cyanotoxin (BMAA) in food on the life span and behavior have been determined [26]. Dietary uptake of BMAA resulted in a substantial accumulation of BMAA in the flies and is associated with a significant reduction in lifespan and age-dependent locomotor activity deficit. *Drosophila* serves as an excellent in vivo model to study the acute and chronic toxicity of BMAA accumulation and can be applied for further mechanistic studies and screening of compounds that may reduce BMAA toxicity or ameliorate neurodegeneration. 

### 3.4. Limitations and Future Directions

In this study, aerosolized HAB water sources with quantifiable BMAA and microcystin toxins were exposed to *Drosophila* and caused chronic damage to their fitness and longevity. However, since the age-dependent vulnerability to aerosolized HAB exposure did not necessarily correlate with toxin concentrations of BMAA and microcystin, other undetected toxins may be present in the collected HAB samples and contribute to the chronic health impact. The two HAB water sources were collected at different locations in South Florida and were cultured in the lab with different durations. HAB-1 was collected in Cape Coral on 2 September 2018 and was used for exposure experiment on 1 May 2019. The material was mostly culture media and the culture mix of phytoplankton. On the other hand, HAB-2 was collected at Lake Okeechobee on 3 March 2019 and was used for an exposure experiment on 3 April 2019 and thus, the water source was relatively fresh and may contain significant amounts of Lake Okeechobee water with a possible host of other organic compounds typical of lake water. The difference between these two collected HAB samples may cause varying short- and long-term impacts on health for different age and gender groups. On the other hand, for negative geotaxis assay and lifespan, both sexes exposed to water in the late-stage groups were doing worse than those exposed to HAB-1 and HAB-2. This could possibly be due to mechanical stress from water with low osmolarity (MilliQ pure water) during the 2 h exposure and age-dependent sensitivity causing different impacts on locomotor functions and longevity for the older flies. 

For future studies, the health impact of aerosolized cyanobacterial toxins other than BMAA and microcystin on *Drosophila* needs to be examined and analyzed. The mechanisms of cyanobacterial toxin-induced neurodegeneration diseases also need to be studied to protect human beings and animals from aerosolized HAB toxins. Examining and analyzing the impact of cyanotoxins provides a powerful and effective bio-indicator, which can guide future prevention for human exposure and reduce the harm to human health.

## 4. Conclusions

The fruit fly, *Drosophila melanogaster*, is an ideal model to analyze the acute and chronic impact on health in vivo. HAB aerosols generated from South Florida water systems pose significant age-dependent health risks, both immediately after exposure and in the form of long-term responses. This study raises a concern about the potential risk to human health following chronic exposure of aerosolized cyanotoxins associated with increasingly frequent HABs in the ocean, coastal waters, and freshwater ecosystems. This study will serve as a basis for the next phase of research to identify specific aerosolized cyanobacterial toxins that mediate major toxicity in health and to examine the mechanisms of cyanobacterial toxin-induced neurodegeneration diseases.

## 5. Materials and Methods 

### 5.1. Aerosol Exposure Apparatus and Aerosol Collection

**Figure 5:**
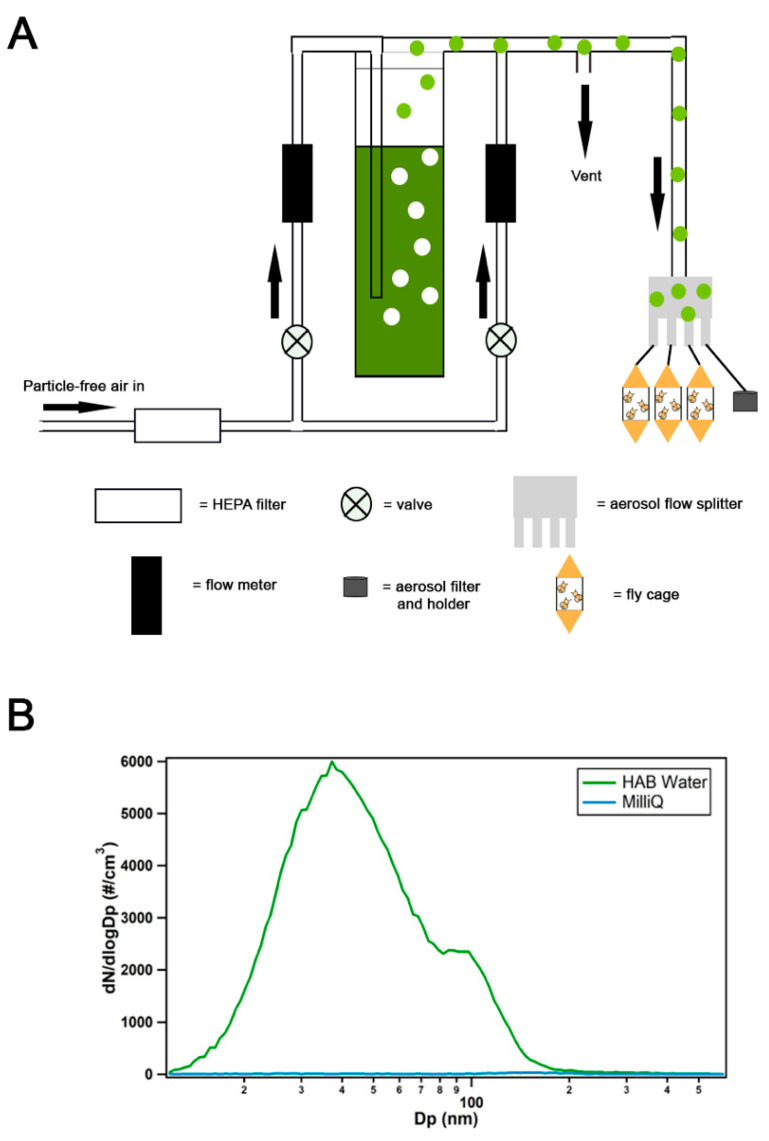
A bubbler apparatus was designed for aerosol exposure and collection. (**A**) A bubbler apparatus used to expose *Drosophila* to aerosolized waters. (**B**) The average particle size distribution of the HAB-3 water (green trace) and the milli-q control experiment (blue line) are shown on a logarithmic scale as dN/dlogDp, where Dp is the electrical mobility diameter.

A bubbler apparatus (Figure 5A) generated lake spray aerosol that was used for fly exposure studies. Particle-free air (Pall, HEPA Capsule Filter) was split between a porosity C (25–50 µm) glass frit used to generate lake spray aerosol and a dilution flow. This is a similar design to other studies on laboratory sea spray aerosol generation [41,42,43]. Before each experiment, the frit was rinsed thoroughly with DI water, acid washed with 10% hydrochloric acid, thoroughly rinsed with milli-q water, wrapped in foil, and combusted at 450 °C for at least 4 h. A flow of 1 Lpm was sent through the glass frit bubbler and combined with an 8–10 Lpm dilution flow. The aerosol-laden flow next entered a four-way aerosol flow splitter. For life span and climbing experiments, the flow was split to three custom-made fly cages containing flies at different life stages—early, young, and late—and a filter cassette holding a 47-mm pre-combusted glass fiber filter (EPM2000), which was used to collect generated aerosols. After each experiment, the filter was wrapped in pre-combusted aluminum foil and placed in a −80 °C freezer until analysis for toxins. Four personal air sampling pumps (Sensidyne Gilian BDX II) were used to pump 2 Lpm across each cage and the aerosol filter.

For the brain morphology experiments, two fly cages were used instead of three to probe the effect of lake spray aerosol on early and late fly life stages. A filter was collected, and aerosol-laden flow in the last line of the flow splitter was sent to a silica gel diffusion drier, then a scanning mobility particle sizer (SMPS, model 3082, TSI Inc., Shoreview, MN, USA) was used to measure particle size distributions from 0.01 to 0.6 µm. A milli-q water control and HAB-3 water culture were used for the brain morphology experiments. The milli-q control produced minimal background counts that were < 2% of those produced by the HAB-3 water (~3000 cm^−3^). Particles generated from the HAB-3 water produced a mode at 38 nm (Figure 5B), similar to previous measurements of lake spray aerosol [34].

### 5.2. Drosophila Culture and Exposure Setup

A laboratory wild type strain of fruit fly, genotype *yw (yellow-white)* was used in the study. Fruit flies were raised on a cornmeal, yeast, and molasses media at room temperature (20 °C) under ambient light. Three age groups of flies were collected at 2 days, 5–10 days, and 20–30 days. Flies at the age of 2 days after eclosion (DAE) are considered as “early-stage”, 5–10 DAE are “young”, and 20–30 DAE are “late-stage” [44]. These three age groups of flies were collected into three screened cages, where one of the openings was sealed with gauze and tape. Each cage contained about 100 flies with around 50 males and 50 females from the same age group. All three cages with three different age groups of flies were exposed to one water source at the same time for 2 h. The group exposed to milli-q water was used as the control.

### 5.3. HAB Water Collection and Toxin Sampling

Three different HAB-containing water sources were used for exposure in this study. The accompanying characteristics of the three HAB water sources are provided in Table 1. The HAB-1 was collected from a canal in Cape Coral, Florida (26°36′21.45′′ N, 81°55′20.20′′ W) containing 555 µg/L of chlorophyll on 2 September 2018, and HAB-2 was collected from Lake Okeechobee (26°49′36.29′′ N, 80°39′59.44′′ W) with 4.3 µg/L of chlorophyll on 3 March 2019. HAB-3 was a monospecific culture of *Microcystis aeruginosa* obtained from a commercial culture collection and maintained under laboratory conditions. Chlorophyll was measured with a calibrated Turner Designs 10-AU Fluorometer (Turner Designs, Mountain View, CA, USA). Toxin concentrations were measured after the filtration of water samples onto pre-combusted glass fiber grade F filters (GF/F, nominal pore size 0.7 μm), extracted using solvents and sonication, and analyzed with high-pressure liquid chromatography triple quadrupole mass spectrometry.

### 5.4. Drosophila Behavior Analysis: Negative Geotaxis Assay

Negative geotaxis assay was performed as previously described [22]. Flies were sexed under carbon dioxide anesthesia on the first day after 2 h of exposure with different water sources. They were maintained in groups of about 20 per each polystyrene vial and transferred to a fresh vial every 3 days. After the first day of exposure, the locomotor activity of the flies was determined by a negative geotaxis assay. A total of ten fruit flies of the same age and same sex were placed in one climbing vial. Negative geotaxis assay was performed half an hour after anesthesia, which is crucial for flies to fully recover from anesthesia. In each climbing apparatus, flies were gently tapped down to the bottom of the vial and the number of fruit flies that climb above 8 cm height in 10 s was counted. Ten trials were performed for each group. The raw data collected from this assay were then converted to a percentage and the average percentage of flies passing the 8 cm height in 10 s in each group over 10 trials was computed using GraphPad Prism (Version 8.1.2, GraphPad Software, San Diego, CA, USA, 2019).

### 5.5. Drosophila Lifespan Assay

Lifespan assay was performed as previously described [27]. A total of 50 newly enclosed flies of each genotype were collected and placed in groups of 10 individuals. Files were kept at 25 °C with 65% humidity, 12 h light and 12 h dark cycles. The number of dead and alive flies were recorded daily after the first day of exposure. Flies dead on the exposure day were not analyzed for overall longevity. The vials were replaced every 1 to 2 days with fresh food. The daily record was continued until every single fruit fly died. The survival curve was graphed using Prism to make a comparison between groups and to analyze the impact of HAB exposure on their longevity.

### 5.6. Immunofluorescence Imaging-Based Morphological Analysis of Drosophila Brain

Brain dissection and fluorescence imaging were carried out to examine brain morphological changes. The brain dissection was performed as previously described [45]. After dissection, brains were fixed with 4% formaldehyde for 10–15 min and washed with PBTx (PBS with 0.4% Triton X-100) three times for 15 min each. Brains were then incubated with primary antibodies overnight in a 4 °C refrigerator. The primary antibody used in this study is mouse anti-BRP (1:250, Developmental Studies Hybridoma Bank, University of Iowa, IA, USA). After being washed three times with PBTx again on the next day, brains were incubated with secondary antibodies overnight. The secondary antibody used in this study is conjugated to Alexa Fluor 555 (1:250, Thermo Fisher Scientific, Waltham, MA, USA). On the third day, 4′,6-diamidino-2-phenylindole (DAPI, 1:300, Invitrogen, Carlsbad, CA, USA), a fluorescent dye, was used to mark nuclei and reveal the overall morphology of the brain. After the vials were rinsed three times with PBTx once more, brains were mounted on the mounting slides with VECTASHIELD Antifade Mounting Medium (Vector Laboratories Inc., Burlingame, CA, USA) and scanned using the Olympus FluoView FV1000 confocal laser scanning fluorescence microscope. Images were processed and analyzed by FluoView 10-ASW Software (Version 4.0a, Olympus, Tokyo, Japan, 2012) and Adobe Photoshop CS6 (Version 19.0, Adobe Inc., San Jose, CA, USA, 2018). The brain size and fluorescence intensity were analyzed afterwards using ImageJ (Version 1.53g, NIH, Bethesda, MD, USA, 2020).

### 5.7. Toxin Analysis

The cyanotoxins BMAA and microcystins were analyzed using high-pressure liquid chromatography triple quadrupole mass spectrometry (HPLC-MS), with a Dionex 3000 HPLC coupled with a ThermoScientific TSQ Altis triple quadrupole mass spectrometer with electrospray ionization interface (Thermo Electron North America LLC, Madison, WI, USA). BMAA analysis used reverse phase chromatography with a Kinetex HILIC column (Phenomenex Inc., Torrence, CA, USA), 150 × 4.6 mm, 5 mm particles, 100 Å, and a 27 min chromatography method with a gradient of water with 0.1% formic acid (eluent A) and acetonitrile with 0.1% formic acid (eluent B); all solvents were HPLC-grade (Fisher Scientific Company LLC, Torrence, IL, USA) [46]. BMAA was detected with positive ion selected reaction monitoring as well as precursor ion scans from 50 to 200 *m/z*. BMAA was identified with the qualitative ion transition 119 to 102 *m/z* (common to other isomers) and quantitative ion transitions 119 to 88 and 76 *m/z*; the isomer L-2,4-diaminobutyric acid dihydrochloride (DAB) was differentiated using the qualitative ion transition 119 to 102 *m/z* and quantitative ion transitions 119 to 101 and 74 *m/z*. Microcystin analysis used reverse phase chromatography with a Kinetex EVO C18 column (Phenomenex Inc., Torrence, CA, USA), 150 × 4.6 mm, 5 mm particles, 100 Å, and a 27 min chromatography method with a gradient of water with 19 mM ammonium hydroxide (eluent A) and 80% methanol with 20% acetonitrile (eluent B); all solvents were HPLC-grade [47]. Microcystins were detected with negative ion selected reaction monitoring with typically three transitions for each congener (common precursor ion). Transitions scanned for the following compounds: microcystin (MC)-LA, MC-LF, MC-LR, MC-LW, MC-LY, MC-RR, MC-WR, MC-YR, and D-Asp-MC-LR. MS settings were determined by direct infusion of commercially available standards for each compound of interest, including BMAA (Sigma Aldrich B107, Saint Louis, MO, USA), DAB (Sigma Aldrich 32830, Saint Louis, MO, USA), and the 9 congeners of microcystin (Enzo Life Sciences, Farmingdale, NY, USA; ALX-350-096, ALX-350-081, ALX-350-012, ALX-250-080, ALX-350-148, ALX-350-043, ALX-350-167, ALX-350-044, ALX-250-173). For each toxin class, water samples were collected on 47 mm combusted glass fiber GF/F filters (nominal pore size 0.7 mm), air samples were collected on 47 mm combusted glass fiber EPM filters. Extraction for BMAA used vortex and sonication in trichloroacetic acid, followed by protein digestion in 6 M hydrochloric acid at 99 °C for 24 h, then sample clean-up using solid phase extraction with Agilent Plexa PCX Bond-Elut cartridges (Agilent Technologies, Santa Clara, CA, USA) [46]. Samples were concentrated to 200 µL and resuspended in a mix of acetonitrile and water with 0.1% formic acid before injection of 20 mL on the HPLC-MS. Prior to extraction, deuterated serine (L-serine-d7, CDN Isotopes, D-6466) was added to each sample as an internal standard; corrections were not made for extraction efficiency, thus final concentrations are a minimum estimate of ng per L of original sample water or air. Extraction for microcystin used vortex and sonication in methanol, particulate material was removed from samples by filtration with 0.2 mm pore size polyethersulfone syringe-tip filters. Prior to extraction leucine enkephalin acetate (ENK, Sigma Aldrich L9133, Saint Louis, MO, USA) was added to each sample as an internal standard; each sample was corrected for extraction efficiency using the final amount of ENK on column per MS run relative to the amount added to the original sample. Samples were concentrated to 200 µL and resuspended in a mix of methanol and water before injection of 50 µL on the HPLC-MS. The limit of quantification (LOQ) for BMAA was approximately 10 pg on column, for microcystins the LOQ varied by congener, ranging from approximately 20 to 80 pg on column, averaging approximately 50 pg on column across the 9 congeners analyzed.

## Figures and Tables

**Table 1:** Physical and chemical characteristics of HAB water sources.

Water Sample	At the Time of Water Sample Collection					At the Time of Exposure Experiment			
Number	Date	Location	Chloro-phyll (μg/L)	Micro-cystin (μg/L)	BMAA (ng/L)	Date	Micro-cystin, water (μg/L)	BMAA, water (ng/L)	Micro-cystin, air (pg/L)
HAB-1	2 September 2018	Cape Coral	555	319.93	<LOQ	1 May 2019	0.37	7.2	<LOQ
HAB-2	3 March 2019	Lake Okeechobee	4.3	N.D.	N.D.	3 April 2019	0.01	1.7	<LOQ
HAB-3	n/a	Monospecific culture of *Microcystis aeruginosa*	n/a	n/a	n/a	9 January 2020	379.38	N.D.	62

Abbreviations: N.D. = not determined; n/a = not applicable; <LOQ = less than the limit of quantification.
